# Authentication of Fennel, Star Anise, and Anise Essential Oils by Gas Chromatography (GC/MS) and Stable Isotope Ratio (GC/IRMS) Analyses

**DOI:** 10.3390/plants13020214

**Published:** 2024-01-12

**Authors:** Brett J. Murphy, Tyler M. Wilson, Emma A. Ziebarth, Christopher R. Bowerbank, Richard E. Carlson

**Affiliations:** D. Gary Young Research Institute, Lehi, UT 84043, USA; bmurphy@youngliving.com (B.J.M.); cbowerbank@youngliving.com (C.R.B.);

**Keywords:** authentication, (*E*)-anethol, essential oil, gas chromatography/mass spectrometry (GC/MS), gas chromatography/isotope ratio mass spectrometry (GC/IRMS)

## Abstract

The aromatic compound (*E*)-anethol is widely used in the flavor, fragrance, and medicinal industries. This compound is commonly produced through steam distillation of fennel, star anise, and anise seed. Given the cost of production, these natural and authentic essential oils are commonly adulterated with lower-cost natural materials or synthetic alternatives. The current study investigates essential oil profiles (gas chromatography/mass spectrometry) and stable isotope ratios (gas chromatography/isotope ratio mass spectrometry) of the abundant compound (*E*)-anethol in both authentic reference standards (*n* = 15) and commercially available samples (*n* = 30). This multifaceted analytical approach establishes techniques for ensuring the authenticity of essential oil sources of (*E*)-anethol and was then used to evaluate the current essential oil market sources of (*E*)-anethol. These findings report that adulteration of (*E*)-anethol-containing natural products takes various forms, and a multifaceted analytical approach is recommended for authentication. Of the commercial samples analyzed for this report, 27% were adulterated.

## 1. Introduction

**Figure 1:**
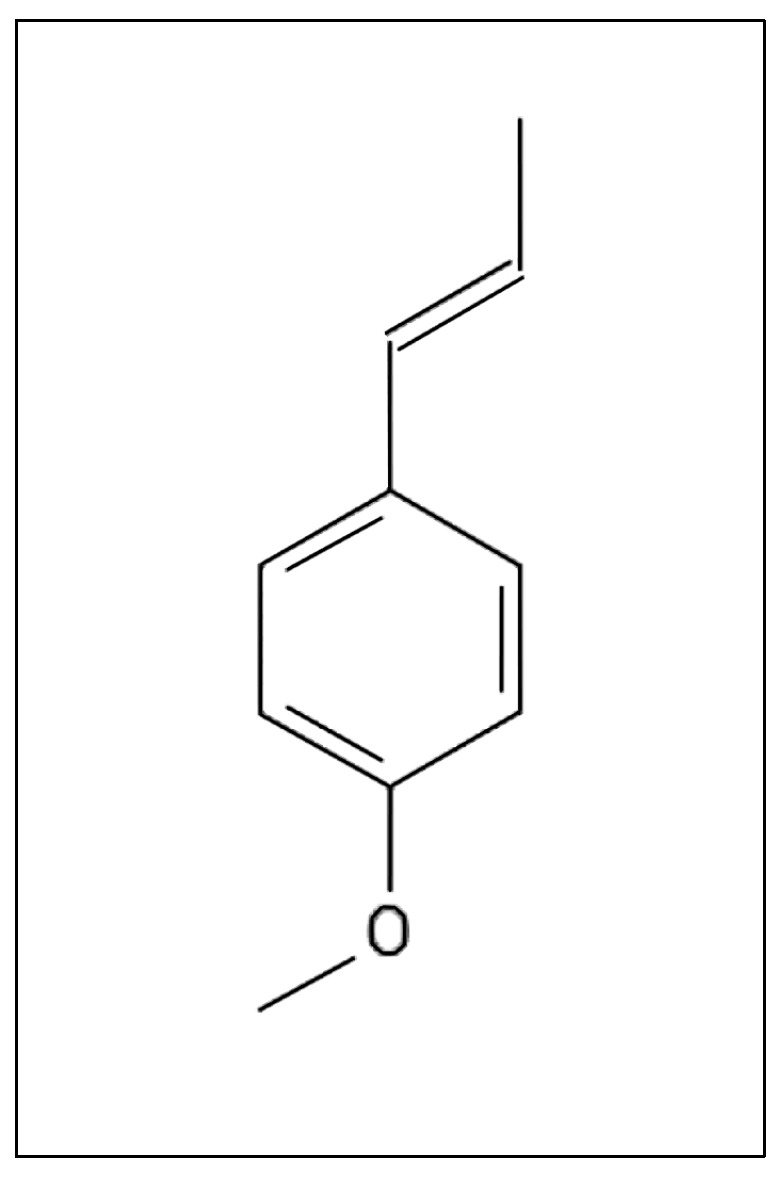
Structure of the compound (*E*)-anethol [8].

(*E*)-anethol is a phenylpropanoid and an important flavoring agent and food ingredient (Figure 1). This compound is commonly used in confectioneries, beverages, personal care products, pharmaceutical flavorings, and natural medicines [1,2,3]. (*E*)-anethol is produced naturally by several plant species and is commonly extracted as the prominent compound from the essential oils of fennel (*Foeniculum vulgare* Mill.), star anise (*Illicium verum* Hook.f.), and anise (*Pimpinella anisum* L.) [4,5,6,7].

**Figure 2:**
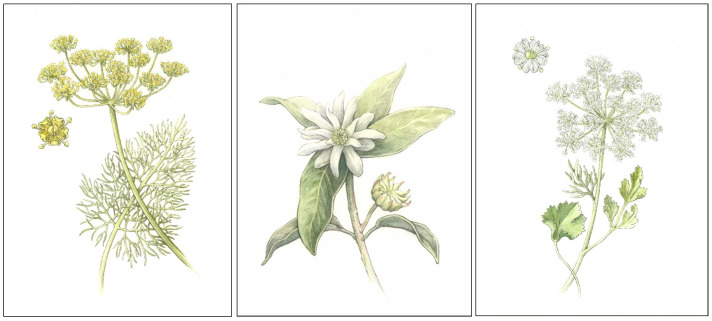
From left to right, botanical illustrations of fennel (*Foeniculum vulgare* Mill.), star anise (*Illicium vulgare* Hook.f.), and anise (*Pimpinella anisum* L.). Plant parts bearing the aromatic seeds are depicted. Botanical illustrations by Zach Nielsen.

Standardized qualities have been defined for fennel (bitter and sweet varieties), star anise, and anise essential oils, which are all distilled from the seeds of each plant species, respectively (Figure 2) [9,10,11,12]. The volatile profile of each essential oil is defined as containing high concentrations of (*E*)-anethol: 50–78% (bitter fennel), 60–80% (sweet fennel), 86–93% (star anise), and 87–94% (anise) [9,10,11,12]. While constituent profiles of the three species are similar, each essential oil contains unique marker compounds that distinguish one from the others. Additionally, profiles may display natural variation, and (*E*)-anethol values may fall outside expected ranges due to any number of abiotic or biotic factors. These factors include cultivation practices, chemotype and provenance of plant, distillation or extraction technique employed, and inherent plant-to-plant variability, among other factors [13,14,15,16,17,18,19,20,21,22,23].

Given the variability of essential oil profiles and the prohibitive costs of natural products, essential oils containing (*E*)-anethol are often adulterated with lower-priced natural or synthetically produced alternatives [3,24,25,26]. To ensure the authentication of natural compounds, gas chromatography/mass spectrometry (GC/MS) and gas chromatography/isotope ratio mass spectrometry (GC/IRMS), among other analytical techniques, have been reported as powerful analytical tools [26,27,28,29,30,31,32]. These same researchers have reported that identification of specific marker compounds by GC/MS and stable isotope analysis of prominent compounds assists in detecting adulteration with synthetic compounds and/or distinguishing natural compounds based on the origin of plant species, chemotype, and provenance.

Two groups of researchers have previously investigated authentication of (*E*)-anethol from fennel and anise by means of chiral analysis and/or GC/IRMS [25,33]. However, both groups found that identifying adulteration and distinguishing the origin of (*E*)-anethol was not always easily performed, as established ranges for natural and synthetic origins somewhat overlapped. The current study also investigates the authenticity of (*E*)-anethol originating from fennel and anise; however, it also incorporates stable isotope data from star anise (a lower-cost natural source of (*E*)-anethol) and essential oil profiles from all three species (GC/MS) and investigates authenticity of commercially available essential oil samples (*n* = 30) from the three species. Findings from the current study confirm previously established stable isotope ratio data and further the field of essential oil authentication, demonstrating that multifaceted analytical approaches are ideal for identifying adulterants in essential oils containing (*E*)-anethol.

## 2. Results

### 2.1. Volatile Compound Profiles (GC/MS)

Authentic essential oils for fennel (*Foeniculum vulgare*) (*n* = 5), star anise (*Illicium verum*) (*n* = 5), and anise (*Pimpinella anisum*) (*n* = 5) were produced by steam distillation and used as reference standards (see Section 4). Additionally, volatile compound reference ranges for these species have been previously established and have identified key compounds that can be used to identify and distinguish the quality of each essential oil [9,10,11,12]. GC/MS analysis of authentic standards (*n* = 15) prepared for this research confirmed the presence of these key marker compounds in each authentic standard, respective to the plant species (Table 1).

Select compounds were identified in authentic essential oils as being unique to each species and present in relatively substantial amounts (≥ 0.2%). As such, these marker compounds can be used in authentication (Table 2) by GC/MS analysis.

### 2.2. Stable Isotope Ratio Analysis (GC/IRMS)

**Figure 3:**
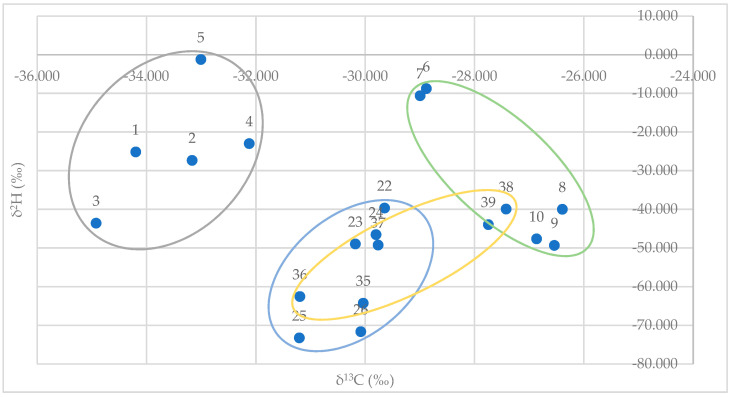
GC/IRMS data plot for δ^2^H (*y*-axis) vs. δ^C^13 (*x*-axis) for synthetic (*E*)-anethol standards (gray; *n* = 5), and authentic essential oils standards of fennel (orange; *n* = 5), star anise (blue; *n* = 5), and anise (green; *n* = 5).

Plotting GC/IRMS data for δ^2^H (*y*-axis) versus δ^13^C (*x*-axis) for synthetic (*E*)-anethol standards (*n* = 5) and authentic standards (*n* = 15) from fennel, star anise, and anise provided a clear distinction from synthetic and authentic/natural samples. There was also a clear distinction between authentic/natural star anise and anise samples. However, there were overlapping values for authentic/natural fennel samples and authentic/natural star anise and anise samples (Figure 3).

Ranges for synthetic samples and authentic/natural samples are provided in Table 3. Stable isotope values, *δ*^2^H and *δ*^13^C, for (*E*)-anethol in commercially available samples are provided in Table 4.

## 3. Discussion

### 3.1. Volatile Compound Profiles (GC/MS)

GC/MS analysis provided the ability to help decipher the origin of the adulteration, such as the addition of other natural sources of (*E*)-anethol and/or the addition of carriers/diluents, such as triethyl citrate or capryl palmitate. 

Fennel sample #45 contained all expected compounds/markers for authentic fennel; however, 43.2% of the composition was triethyl citrate. Fennel sample #47 lacked the authentic fennel marker (fenchone) and contained markers for star anise (0.9% linalool, 0.1% α-terpineol, 1.7% foeniculine) as well as capryl palmitate (35.4%). Star anise sample #31 contained all expected compounds/markers for authentic star anise; however, 36.5% of the composition was triethyl citrate. Anise sample #12 contained unexpected compounds (2.8% linalool, 2.8% menthol) and lacked markers for authentic anise (γ-himachalene, pseudoisoeugenyl-2-methylbutyrate). Anise sample #18 contained unexpected compounds (0.2% α-terpineol, 72.8% triethyl citrate). Anise sample #19 contained unexpected compounds (1.9% linalool, 1.6% α-terpineol) and lacked markers for authentic anise (γ-himachalene, pseudoisoeugenyl-2-methylbutyrate). 

Considering GC/MS data alone, 20% of the commercially obtained essential oil samples analyzed (two fennel, one star anise, three anise) were adulterated. Adulteration of commercially available essential oil samples (6 of 30 samples) likely occurred using two different forms: the addition of carriers/diluents in four samples (anise #18; star anise #31; fennel samples #45 and 47) and the addition of other natural or synthetic sources of (*E*)-anethol in four samples (anise samples #12, 18, and 19; fennel #47). While GC/MS is a powerful tool in detecting/identifying the addition of carriers/diluents, it is not always ideal as a standalone tool for distinguishing the source of the unexpected/additional volatile compounds in samples. For example, fennel sample #47 appears to be “extended” with star anise and a carrier/diluent (lack of natural fennel markers, all star anise markers present, addition of capryl palmitate), but anise samples #12, 18, and 19 were less conclusive as to how they were likely adulterated. 

### 3.2. Stable Isotope Ratio Analysis (GC/IRMS)

When evaluating the authenticity of commercially available samples, stable isotope data are less conclusive than GC/MS data. If only considering stable isotope data, 83% of commercially available samples fall outside established ranges (Table 3 and Table 4). However, this is largely due to overlapping δ^2^H values (Table 3) and unexpected values lower than −73.262 (Table 4). Taking this into consideration, δ^2^H values alone do not appear to be a reliable measurement for (*E*)-anethol authentication. Given that both fennel and anise are cultivated throughout the world and at various elevations, the inability to directly correlate δ^2^H values with authenticity is likely due to the inherent association of δ^2^H values with ocean water and storm patterns and the distance of cultivation from the ocean [32]. These δ^2^H findings appear to be consistent with those of previous researchers such that values from the current study and data from the other two research groups [25,33] all appear to overlap (values for synthetic vs. natural sources of (*E*)-anethol) and are somewhat unreliable for authentication (Table 5). However, that conclusion could change, given additional reference standards of known origin and cultivation practices. Stable isotope data for δ^13^C from the current study are relatively consistent with findings from both research groups and appear to be a reliable tool for authentication.

### 3.3. Multifaceted Approach

Literature published in the 1950s stated that anise essential oil was valued higher than fennel and star anise in the flavor and fragrance industries [24]. This same literature indicates that anise essential oil was often adulterated with (*E*)-anethol from other natural sources or from synthetic (*E*)-anethol. While current prices for these essential oils or synthetic (*E*)-anethol are much higher than those from the 1950s, price-point trends appear to be the same (private communication; priced highest to lowest: anise, fennel, star anise, synthetic (*E*)-anethol).

As was mentioned previously, GC/MS data suggested that six samples (#12, 18, 19, 31, 45, and 47) were adulterated. Based on the adulteration technique likely employed in these commercially available samples, stable isotope data (*δ*^2^H and *δ*^13^C) confirmed and supported the GC/MS data and findings (Table 3 and Table 4). Additionally, *δ*^13^C provided evidence that anise samples #12, 18, and 19 were adulterated with synthetic (*E*)-anethol (Table 3 and Table 4). Considering these data alongside the GC/MS data, which showed only the presence of some but not all markers for other natural and cheaper sources of (*E*)-anethol, suggests that a synthetic source of (*E*)-anethol was likely used.

*δ*^13^C data also suggest that two additional samples (star anise #29, fennel #46) contained synthetic (*E*)-anethol (Table 3 and Table 4). Given that both samples contained markers for natural star anise and fennel, respectively, these samples may have an addition of synthetic (*E*)-anethol to “extend” the samples. Given economic incentives to adulterate and falsely market essential oils, the current study concludes that 8 of the 30 (27%) commercially available fennel, star anise, and anise essential oil samples were adulterated. Adulteration took the form of the addition of carriers/diluents, the use of cheaper natural sources of (*E*)-anethol (i.e., star anise), and/or the use of synthetic (*E*)-anethol. While GC/MS and *δ*^13^C stable isotope data proved useful in identifying adulteration, *δ*^2^H stable isotope data did not. However, *δ*^2^H data may prove useful, given the addition of more authentic reference standards.

## 4. Materials and Methods

Synthetic (*E*)-anethol commercial reference samples (*n* = 5) were purchased from various retailers (TCI America, Division of Tokyo Chemical Industry, Portland, OR, USA; MilliporeSigma, Sigma-Aldrich, St. Louis, MS, USA; Acros Organics, Janssen-Pharmaceuticalaan, Geel, Belgium). *Pimpinella anisum* (anise), *Foeniculum vulgare* (fennel), and *Illicium verum* (star anise) seeds were procured directly from farmed sources or online retailers for in-house steam distillation and creation of authentic reference standards (*n* = 15). Additionally, anise, fennel, and star anise essential oil samples (*n* = 30) were procured from in-store and online retailers to investigate the authenticity of commercially available samples. For simplicity and consistency, samples were referred to by a number from 1 to 50 (Table 6). All reference samples and commercially available essential oil samples were stored at room temperature, as received in their original sealed amber glass bottle, until analysis.

**Figure 4:**
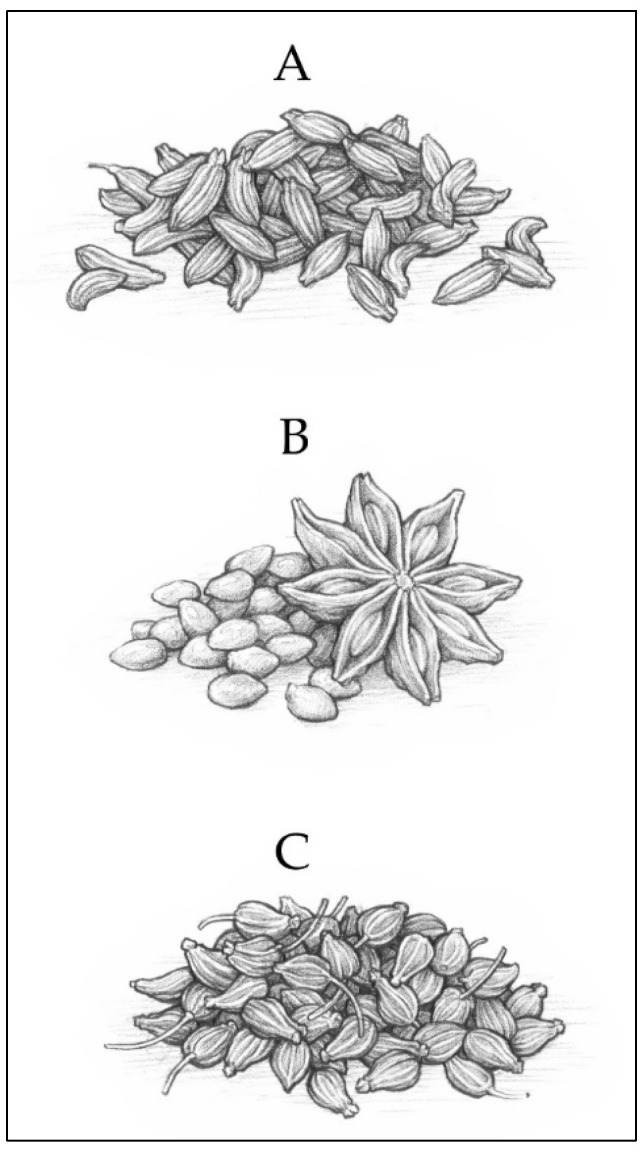
Botanical illustration of (**A**) fennel, (**B**) star anise, and (**C**) anise seeds. Botanical illustrations by Zach Nielsen.

Laboratory-scale distillation for authentic in-house standards was as follows: 1.5 L of water was added to a 2 L steam generator that fed into a 2 L distillation chamber. The plant material of each species (seeds) was ruptured to increase surface area (Figure 4), then accurately weighed and added to the distillation chamber. Distillation was performed for 1.5 h from passover by indirect steam, and essential oil was separated by a cooled condenser and Florentine flask. Essential oil samples were each filtered and stored at room temperature in a sealed amber glass bottle until analysis.

Essential oil samples were analyzed, and volatile compounds were identified and quantified by GC/MS using an Agilent 7890B GC/5977B MSD (Agilent Technologies, Santa Clara, CA, USA) and Agilent J&W DB-5, 60 m × 0.25 mm, 0.25 μm film thickness, fused silica capillary column. Operating conditions: 0.1 μL of sample (20% soln. for essential oils in ethanol), 100:1 split ratio, initial oven temp. of 40 °C with an initial hold time of 5 min, and oven ramp rate of 4.5 °C per min to 310 °C with a hold time of 5 min. The electron ionization energy was 70 eV, scan range 35–650 amu, scan rate 2.4 scans per s, source temp. 230 °C, and quadrupole temp. 150 °C. Compounds were identified using the Adams volatile oil library [34] using a Chemstation library search in conjunction with retention indices. Note that *p*-anis aldehyde/(*Z*)-anethol elutes as a single peak. Their amounts were determined by the ratio of masses 107 and 135 (*p*-anis aldehyde), 117 and 148 ((*Z*)-anethol). Additionally, compound retention time was verified using reference compounds (MilliporeSigma, Sigma-Aldrich, St. Louis, MO, USA).

The hydrogen and carbon stable isotope ratios of essential oils were analyzed by GC/IRMS using a Thermo TRACE 1310 GC coupled to a Thermo Delta V Advantage Isotope Ratio Mass Spectrometer (ThermoFisher Scientific, Waltham, MA, USA) with an Agilent J&W DB-5, 0.25 mm × 60 m, 0.25 μm film thickness, fused silica capillary column.

Essential oil samples were prepared for GC/IRMS analysis as follows: 35 mg of sample was weighed into a 2 mL transparent glass vial and brought up to 1 mL with hexane. A 100 μL aliquot was placed into a second vial, which was then brought up to 1 mL with hexane and used for ^2^H/^1^H analysis. From the second sample vial, a 90 μL aliquot was removed and placed into a third vial, brought to 1 mL in hexane, and used for ^13^C/^12^C analysis.

GC/IRMS operating conditions were as follows: splitless injection of 1 μL of sample with splitless time set at 0.25 min, injection port 270 °C, initial oven temp. 50 °C with an initial hold time of 2.0 min, oven ramp rate of 6.0 °C per min to 250 °C with a hold time of 2.0 min, then an oven ramp rate of 10.0 °C per minute to 310 °C with a hold time of 7.0 min, and helium carrier gas with constant flow 1.55 mL/min. After passing through the capillary column, samples were sent through the HTC reactor for ^2^H/^1^H analysis or the combustion reactor for ^13^C/^12^C analysis. HTC reactor temp. was set to 1420 °C and was regularly conditioned by injecting 1 μL of hexane in backflush mode. The combustion reactor temp. was set to 1000 °C and was conditioned with oxygen at regular intervals.

To normalize IRMS results, reference materials were purchased from Dr. Arndt Schimmelmann at Indiana University (Bloomington, IN, USA) and from the United States Geological Survey (USGS)—Reston Stable Isotope Laboratory. *δ*^2^H isotope ratios are expressed relative to VSMOW and *δ*^13^C isotope ratios to VPDB. The following three reference materials, along with their known values, were used to normalize results: hexadecane #C (USGS69), *δ*^2^H: 381.4‰, *δ*^13^C: −0.57‰; nonadecane #2, *δ*^2^H: −56.3‰, *δ*^13^C: −31.99‰; and tetradecanoic acid methyl ester #14M, −231.2‰, *δ*^13^C: −29.98‰.

Samples were analyzed in triplicate to ensure repeatability. *δ*^2^H values are reported with a standard deviation ≤ 2.0‰ and *δ*^13^C values are reported with a standard deviation ≤ 0.2‰.

## 5. Conclusions

Previous studies on the authentication of natural essential oil sources of (*E*)-anethol (fennel and/or anise) relied heavily on stable isotope data, with data being somewhat inconclusive. In the current study, star anise essential oil samples, in addition to fennel and anise, were investigated. Despite the addition of another common and natural essential oil source of (*E*)-anethol in the current study, stable isotope data, when considered alone, were still somewhat inconclusive. However, using a multifaceted analytical approach with both gas chromatography/mass spectrometry (GC/MS) and gas chromatography/isotope ratio mass spectrometry (GC/IRMS) proved useful. Upon analyzing commercially available essential oil samples of fennel, star anise, and anise (*n* = 30) by GC/MS, 6 of the 30 (20%) appeared to be adulterated. Of the six adulterated samples, a definitive understanding of the source of adulteration was clear in three of the samples (the addition of carriers/diluents and the use of star anise essential oil when the label claimed fennel was used). While three of the adulterated commercially available anise essential oil samples contained some unexpected compounds (linalool and/or α-terpineol, both markers of star anise), the exact source of adulteration was not deciphered by GC/MS alone. This may be partially explained by the fact that both linalool and α-terpineol are common compounds in many essential oils, possibly even in other authentic/natural anise samples, and that they only act as markers of star anise when in association with the third compound previously mentioned, foeniculine. GC/IRMS (*δ*^13^C) provided clarification here, that these three adulterated anise essential oil samples contained synthetic sources of (*E*)-anethol. GC/IRMS (*δ*^13^C) also suggested the adulteration of two other commercially available essential oil samples (increasing the total adulterated samples from 20% to 27%) by the use of synthetic (*E*)-anethol; these were samples that otherwise did not contain any detectable unexpected markers (GC/MS). Using GC/MS and GC/IRMS together proved to be a powerful tool in both detecting adulteration of natural essential oil sources of (*E*)-anethol and determining the method of adulteration.

One of the adulterated anise samples (#18) in this study contained the expected natural markers for anise (γ-himachalene, pseudoisoeugenyl-2-methylbutyrate) as well as one of the markers for star anise (α-terpineol), suggesting that this anise sample was “extended” with a cheaper source of (*E*)-anethol (possibly star anise). The approach in the current study resulted in both the detection of adulteration in samples and in determining the method of adulteration, but not the extent of adulteration. Future studies could create “self-adulterated” samples at various ratios to calculate what percent of the sample is original/authentic and what percent adulterated in these samples where “extension” occurs. This approach would also provide data for determining to which extent/level adulteration can be detected by analytical techniques.

Future studies should also contain a larger group of both synthetic (*E*)-anethol standards and authentic reference standards of fennel, star anise, and anise essential oils. A larger group of samples will strengthen conclusions as well as add clarity to stable isotope values and ranges, particularly with *δ*^2^H data.

## Figures and Tables

**Table 1:** Established ranges of key compounds by the International Organization for Standardization (ISO) for the oils of bitter fennel (ISO 17412) [9], star anise (ISO 11016) [11], and anise (ISO 3475) [12] and by the Association Française de Normalisation (AFNOR) for sweet fennel (NF T75-257) [10]. Average values (GC/MS) from authentic standards of fennel (*n* = 5), star anise (*n* = 5), and anise (*n* = 5) provided. Values less than 0.1% are denoted as trace (tr) and those not detected (nd). For compounds not included in the ISO or AFNOR standards, “n/a” is denoted. KI is the Kovat’s Index value and was previously calculated by Robert Adams using a linear calculation on a DB-5 column [34].

Compound Name	KI	Bitter Fennel (ISO 17412) [9] ^1^	Sweet Fennel (NF T75-257) [10]	Star Anise (ISO 11016) [11]	Anise (ISO 3475) [12]	Fennel (auth. std.) avg. Value (*n* = 5)	Star Anise (auth. std.) avg. Value (*n* = 5)	Anise (auth. std.) avg. Value (*n* = 5)
		area%						
α-pinene	932	2.0–11.0	1.0–8.0	0.1–1.5	n/a	3.7	0.6	tr
β-pinene	974	tr-1.0	nd-1.0	n/a	n/a	0.2	tr	nd
myrcene	988	0.5–2.0	nd-1.5	n/a	n/a	0.3	tr	tr
α-phellandrene	1002	tr-8.5	0.2–5.0	nd-0.7	n/a	0.4	0.1	nd
limonene	1024	1.0–6.0	1.0–8.0	0.2–6.0	n/a	2.3	3.4	tr
γ-terpinene	1054	n/a	nd-1.5	n/a	n/a	0.5	tr	tr
fenchone	1083	10.0–25.0	8.0–20.0	n/a	n/a	18.5	nd	nd
linalool	1095	n/a	n/a	0.2–2.5	n/a	nd	0.5	nd
α-terpineol	1186	n/a	n/a	nd-0.3	n/a	nd	0.2	nd
methyl chavicol	1195	1.0–6.0	2.0–6.0	0.6–6.0	0.5–3.0	17.0	3.1	3.7
*p*-anis aldehyde	1247	tr-1.0	nd-2.0	0.1–0.5	0.1–1.4	2.1	3.1	1.2
(*Z*)-anethol	1249	nd-0.5	nd-0.5	0.1–1.0	0.1–0.6	0.2	0.1	0.1
(*E*)-anethol	1282	50.0–78.0	60.0–80.0	86.0–93.0	87.0–94.0	52.1	86.1	93.3
anisyl methyl ketone	1380	nd-1.0	n/a	n/a	n/a	1.7	tr	nd
*cis*-α-bergamotene	1411	n/a	n/a	0.04–0.09	n/a	nd	0.1	nd
(*E*)-caryophyllene	1417	n/a	n/a	nd-0.8	n/a	nd	0.1	nd
*trans*-α-bergamotene	1432	n/a	n/a	0.06–0.6	n/a	nd	0.1	nd
γ-himachalene	1481	n/a	n/a	n/a	1.0–5.0	nd	nd	0.8
foeniculine	1677	n/a	n/a	0.1–3.0	n/a	nd	1.0	nd
pseudoisoeugenyl-2-methylbutyrate	1842	n/a	n/a	n/a	0.3–2.0	nd	nd	0.5

^1^ (*E*)-anethol type profile.

**Table 2:** Volatile compounds identified as unique markers in the essential oils (reference standards) of fennel, star anise, or anise. The compound name, KI, and if the compound was detected or not detected (nd) are indicated. KI is the Kovat’s Index value and was previously calculated by Robert Adams using a linear calculation on a DB-5 column [34].

Compound Name	KI	Fennel	Star Anise	Anise
fenchone	1083	detected	nd	nd
linalool	1095	nd	detected	nd
α-terpineol	1186	nd	detected	nd
γ-himachalene	1481	nd	nd	detected
foeniculine	1677	nd	detected	nd
pseudoisoeugenyl-2-methylbutyrate	1842	nd	nd	detected

**Table 3:** Stable isotope ratios, *δ*^2^H and *δ*^13^C, for (*E*)-anethol samples: synthetic (*n* = 5), authentic fennel (*n* = 5), authentic star anise (*n* = 5), and authentic anise (*n* = 5). Samples were analyzed in triplicate to ensure repeatability (*δ*^2^H values are reported with a standard deviation ≤ 2.0‰ and *δ*^13^C values are reported with a standard deviation ≤ 0.2‰). *δ*^2^H isotope ratios are expressed relative to VSMOW and *δ*^13^C isotope ratios to VPDB.

Sample Type	(*E*)-Anethol	
	*δ*^2^H (‰)	*δ*^13^C (‰)
Synthetic standards	−43.618 to −1.280	−34.921 to −32.120
Authentic fennel	−64.282 to −39.964	−31.194 to −27.420
Authentic star anise	−73.262 to −39.709	−31.203 to −29.644
Authentic anise	−49.351 to −8.808	−28.993 to −26.392

**Table 4:** Stable isotope ratios, *δ*^2^H and *δ*^13^C, for (*E*)-anethol prominent commercially available samples (*n* = 30): anise (11–21), star anise (27–34), and fennel (40–50). Samples were analyzed in triplicate to ensure repeatability (*δ*^2^H values are reported with a standard deviation ≤ 2.0‰ and *δ*^13^C values are reported with a standard deviation ≤ 0.2‰). *δ*^2^H isotope ratios are expressed relative to VSMOW and *δ*^13^C isotope ratios to VPDB.

Commercial Sample Reference Number	(*E*)-Anethol	
	*δ*^2^H (‰)	*δ*^13^C (‰)
11	−45.028	−28.510
12	−65.342	−34.231
13	−52.736	−28.646
14	−61.008	−30.096
15	−62.249	−27.873
16	−77.953	−28.296
17	−43.643	−27.777
18	−49.309	−32.219
19	−46.709	−33.169
20	−27.548	−30.743
21	−62.233	−26.665
27	−74.102	−30.642
28	−76.371	−29.440
29	−50.539	−32.231
30	−103.375	−29.709
31	−116.542	−29.504
32	−62.419	−29.570
33	−107.085	−30.385
34	−90.776	−27.454
40	−66.107	−28.186
41	−41.512	−29.438
42	−48.991	−27.436
43	−99.516	−29.016
44	−101.654	−28.964
45	−102.824	−30.831
46	−54.368	−32.481
47	−101.402	−30.925
48	−95.312	−28.709
49	−78.596	−29.941
50	−63.532	−28.978

**Table 5:** Stable isotope ratios, *δ*^2^H and *δ*^13^C, for (*E*)-anethol samples: synthetic, authentic fennel, and authentic anise from 3 research groups (current study, Greule and associates [25], Bilke and associates [33]). For all research groups, *δ*^2^H isotope ratios are expressed relative to VSMOW and *δ*^13^C isotope ratios to VPDB. When values were not determined by researchers, “n/a” is denoted.

Sample Type	Data Source	(*E*)-Anethol	
		*δ*^2^H (‰)	*δ*^13^C (‰)
Synthetic standards	Current Study	−43.618 to −1.280	−34.921 to −32.120
	Greule et al. [25]	−150.9 to −61.5	−33.14 to −27.35
	Bilke et al. [33]	−79 to −20	−32.1 to −24.8
Authentic fennel	Current Study	−64.282 to −39.964	−31.194 to −27.420
	Greule et al. [25]	−114.3 to −79.8	−31.82 to −27.05
	Bilke et al. [33]	−84 to −67	−28.3 to −26.6
Authentic anise	Current Study	−49.351 to −8.808	−28.993 to −26.392
	Greule et al. [25]	n/a	n/a
	Bilke et al. [33]	−74 to −46	−26.3 to −25.3

**Table 6:** Reference and essential oil sample (*n* = 50) details, including sample reference number, sample name, country/region of origin, and sample type. When information was not available, “n/a” is denoted.

Sample Number	Sample Name	Country of Origin	Sample Type
1	(*E*)-anethol standard	n/a	synthetic standard
2	(*E*)-anethol standard	n/a	synthetic standard
3	(*E*)-anethol standard	n/a	synthetic standard
4	(*E*)-anethol standard	n/a	synthetic standard
5	(*E*)-anethol standard	n/a	synthetic standard
6	anise	Egypt	in-house standard
7	anise	Egypt	in-house standard
8	anise	Turkey	in-house standard
9	anise	Turkey	in-house standard
10	anise	Spain	in-house standard
11	anise	Spain	commercial sample
12	anise	unknown	commercial sample
13	anise	Spain	commercial sample
14	anise	Spain	commercial sample
15	anise	Spain	commercial sample
16	anise	Spain	commercial sample
17	anise	Spain	commercial sample
18	anise	unknown	commercial sample
19	anise	India	commercial sample
20	anise	Spain	commercial sample
21	anise	unknown	commercial sample
22	star anise	China	in-house standard
23	star anise	China	in-house standard
24	star anise	China	in-house standard
25	star anise	Vietnam	in-house standard
26	star anise	Vietnam	in-house standard
27	star anise	China	commercial sample
28	star anise	China	commercial sample
29	star anise	unknown	commercial sample
30	star anise	China	commercial sample
31	star anise	unknown	commercial sample
32	star anise	Vietnam	commercial sample
33	star anise	China	commercial sample
34	star anise	unknown	commercial sample
35	fennel	Europe	in-house standard
36	fennel	Europe	in-house standard
37	fennel	Sicily	in-house standard
38	fennel	Tasmania	in-house standard
39	fennel	Tasmania	in-house standard
40	fennel	unknown	commercial sample
41	fennel	Tasmania	commercial sample
42	fennel	Tasmania	commercial sample
43	fennel	Spain	commercial sample
44	fennel	Spain	commercial sample
45	fennel	unknown	commercial sample
46	fennel	Slovenia	commercial sample
47	fennel	unknown	commercial sample
48	fennel	unknown	commercial sample
49	fennel	unknown	commercial sample
50	fennel	Tasmania	commercial sample

## Data Availability

The data presented in this study are available upon request from the corresponding author.
